# The formation of chromatin domains involves a primary step based on the 3-D structure of DNA

**DOI:** 10.1038/s41598-018-35851-0

**Published:** 2018-12-13

**Authors:** Giorgio Bernardi

**Affiliations:** 1 Science Department, Roma Tre University, Viale Marconi 446, 00146 Rome, Italy; 2 Stazione Zoologica Anton Dohrn, Villa Comunale, 80121 Naples, Italy

## Abstract

The general model presented here for the formation of chromatin domains, LADs and TADs, is primarily based on the 3-D structures of the corresponding DNA sequences, the GC-poor and GC-rich isochores. Indeed, the low-heterogeneity GC-poor isochores locally are intrinsically stiff and curved because of the presence of interspersed oligo-Adenines. In contrast, the high-heterogeneity GC-rich isochores are in the shape of peaks characterized by increasing levels of GC and of interspersed oligo-Guanines. In LADs, oligo-Adenines induce local nucleosome depletions leading to structures that are well suited for the attachment to (and embedding in) the lamina. In TADs, the gradients of GC and of oligo-Guanines are responsible for a decreasing nucleosome density, decreasing supercoiling and increasing accessibility. This “moulding step” shapes the “primary TADs” into loops that lack self-interactions, being CTCF/cohesin-free structures. The cohesin complex then binds to the tips of “primary TADs” and slides down the loops, thanks to Nipbl, an essential factor for loading cohesin and for stimulating its ATPase activity and its translocation. This “extruding step” leads to closer contacts and to self-interactions in the loops and stops at the CTCF binding sites located at the base of the loops that are thus closed and insulated.

## Introduction

Fifty years ago, well before any sequencing technique was available, a new approach, later called compositional genomics, was developed in order to understand the organization of the mammalian genome. This approach was based on the “short-sequence designs”^1^ of DNA molecules. Since short sequences are responsible for (1) the fine structure of the double helix; and (2) the interactions of DNA with proteins, compositional genomics is, in fact, based on genome structure and function.

The original investigation^2^ concerned two satellite DNAs, that appeared in CsCl density gradient ultracentrifugation experiments as unresolved shoulders on the “main band” DNAs of mouse and guinea pig, respectively, and that were completely separated by ultracentrifugation in Cs_2_SO_4_/Ag^+^ gradients. The separation was possible because of the differential binding of silver ions on the short repeats of the satellite DNAs and led to the conclusion that satellites DNA are “conformationally slightly different” from main-band DNA, the differences being related to their particular nucleotide sequences^2^. This point was confirmed by the different compositions of the short-sequences of the two satellites specifically split by DNAses^3^. When the Cs_2_SO_4_/Ag^+^ was applied to the bovine genome, the standard mammalian genome at that time, not only several cryptic satellite DNAs were revealed, but, much more importantly, the “main band” DNA molecules were resolved into three families or “major components”^4^. This was the first evidence that, neglecting satellites, the genome consisted of large DNA regions that differed in the frequency of short-sequence binding sites for silver ions and of short-sequences split by DNAses. The “major components” of mammalian DNAs were shown to be “fairly homogeneous” sequences originally estimated as ≥0.3 Mb in size^5,6^ that were called isochores^7^. Subsequent investigations showed that isochores from the human genome (and other mammalian genomes) belong to five families, L1, L2, H1, H2, H3, characterized by increasing GC levels, increasing compositional heterogeneity, increasing gene density and by DNA sizes ≥0.2 Mb. These results were confirmed when sequences from the human genome became available^8–10^ (for a review, see ref.^11^).

While the functional importance of isochores was already evident a long time ago because of their correlations with all genome properties tested and led to their definition as “a fundamental level of genome organization”^12^, the basic reason for their existence is not yet understood^12^, even if links with DNA and chromatin structure were predicted^13^ and later shown. Indeed, the GC-rich, gene-rich, and the GC-poor, gene-poor isochores (the “genome core” and the “genome desert”, see Supplementary Table S1 and Fig. S1) were shown to correspond at interphase to “open” and “closed” chromatins (later called A and B compartments^14^) that were centrally and peripherally located, respectively, in the nucleus^15^.

Recent investigations^16^ showed that maps of GC-rich and GC-poor isochores of all human and mouse chromosomes match maps of TADs, the Topologically Associating Domains, 0.2–2 Mb in size^17^, and maps of LADs, the Lamina Associated Domains, ~0.5 Mb medium size^18^ (that are characterized by histone modificator H3K27me3) respectively (see the Nomenclature section in Materials and Methods). In fact, the average size, ~0.9 Mb, of human isochores^10^, is almost identical to that, ~0.88 Mb, of 91% of the mouse chromatin domains^19^, and both isochores and chromatin domains are evolutionarily conserved in mammals^11,19^. A work still in press showed that the isochores of *Drosophila*^20^ also correspond to TADs^21^.

It should be noted that the current investigations on chromatin based on the Hi-C approach have not taken into account earlier observations on isochores that could provide critical insights. This was done here by studying isochores not only in their composition but also in their topology, and led to the discovery that isochores are characterized by 3-D structures that play a crucial role in the “moulding” of TADs and LADs.

## Results and Discussion

### A working hypothesis

**Figure 1:**
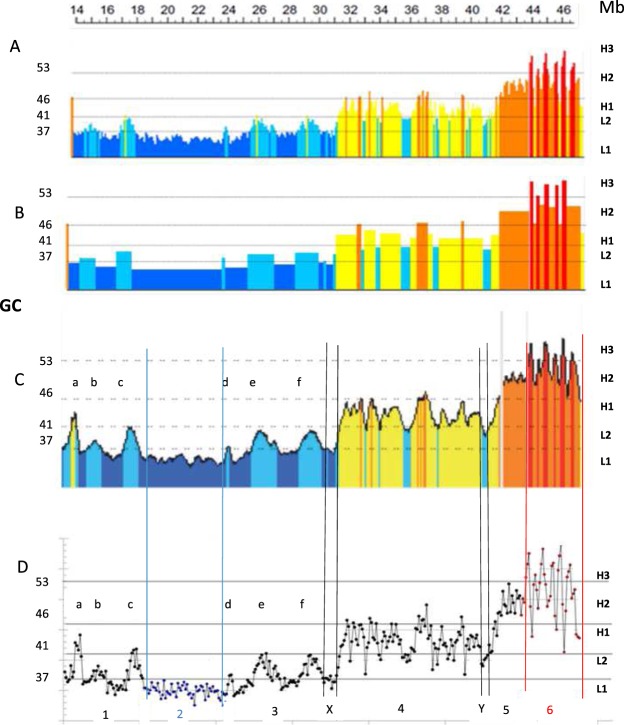
Compositional and isochore profiles of human chromosome 21. (**A**) Compositional profile of release hg38 as seen through non-overlapping 100-Kb windows^46^. DNA sequences from isochore families L1 to H3 are represented in different colors, deep blue, light blue, yellow, orange, red, respectively. The left-side ordinate values are the minima GC values between the isochore families (see Supplementary Table S1). (**B**) Isochore profile (release hg38) using a non-overlapping 100Kb window and the isoPlotter program^46^ with “fixed” isochore borders (see Supplementary Table S1). (**C)** Isochore profile (from the matched b37 assembly^47^) using a sliding window approach with “fixed” isochore borders (see Supplementary Table S1). This profile is slightly more extended on the centromeric (left) side than those of (**A**,**B**). Permissions to publish (**A**–**C**) were obtained from the copyright owners. (**D**) GC levels of 100 Kb windows are presented as a point-by-point plot. This figure shows that, except for the H1 (a) and L2^+^ peaks (b to f), that happen to be more abundant in chromosome 21 compared to other chromosomes, individual isochores from the H1 to H3 families are in the form of peaks that do not appear in a clear way in the standard presentation of compositional profiles of chromosomes (**A**). Black, blue and red lines, as well as double lines (X,Y), separate regions 1 to 6.

The starting point of this work was the consideration that the match between isochores and chromatin domains^16^ suggested a possible similarity between the 3-D structures of the low-heterogeneity GC-poor and the high-heterogeneity GC-rich isochores on the one hand, and the compositionally even structures of LADs and the loops of TADs, on the other. This working hypothesis was tested on human chromosome 21, which comprises isochores from all the five families and, being the smallest human chromosome, allows a more expanded graphical presentation of data. This was done by comparing the previous results obtained by the compositional approach used so far with the new results derived from a topological approach. The previous results basically comprised (1) the compositional profile of the DNA sequence as obtained through non-overlapping 100Kb windows (Fig. 1A; see Materials and Methods for the choice of this window size); and (2) the assemblies of the 100Kb sequences into isochores using a fixed window (Fig. 1B) or a sliding-window approach (Fig. 1C). The new results, in contrast, rely on a point-by-point plot of the GC levels of 100Kb DNA segments.

### The 3-D structure of isochores

**Figure 2:**
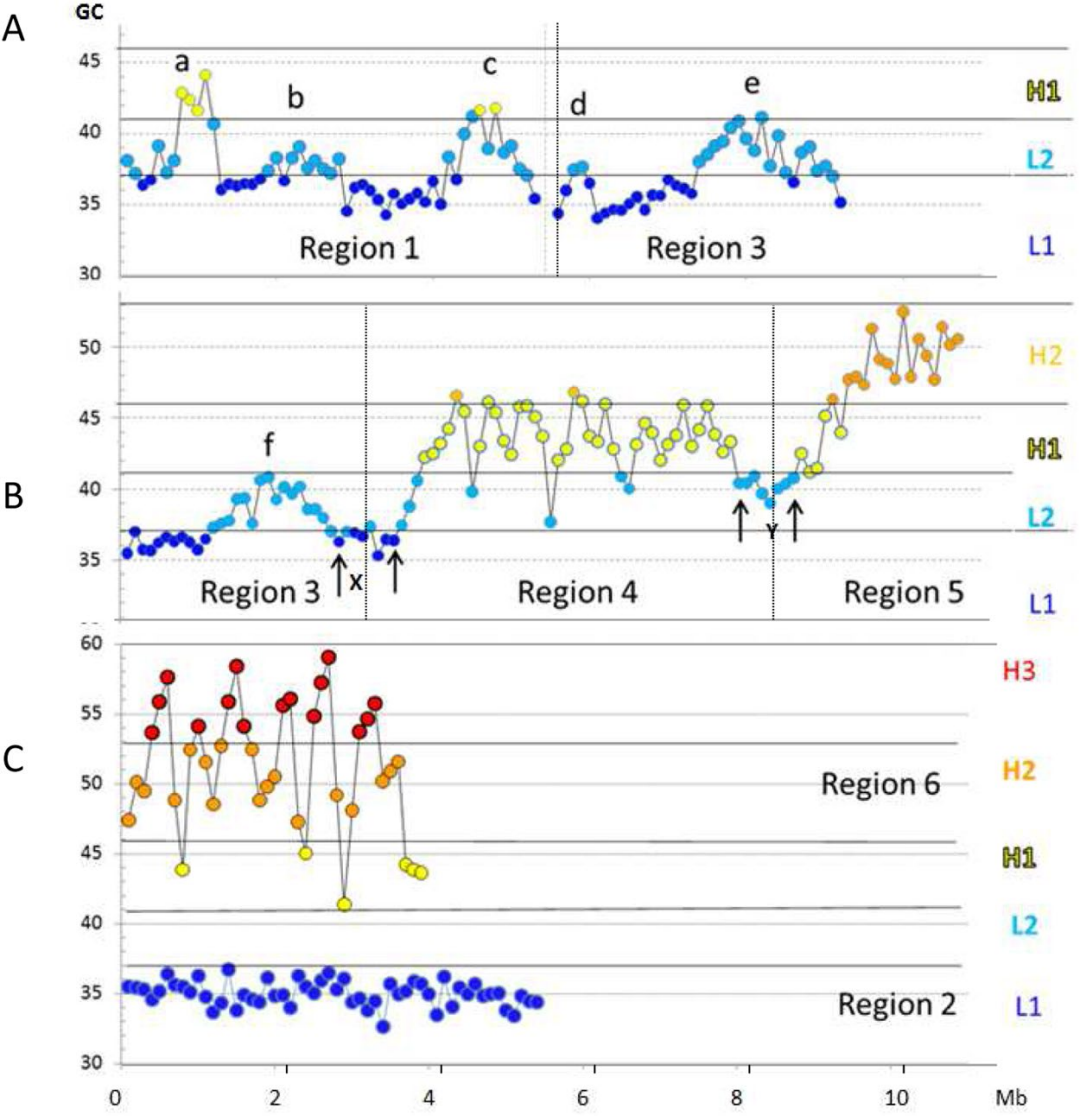
Figure 1D as displayed at a higher magnification. Thick horizontal lines correspond to minima GC values between isochore families. The color code is as in Fig. 1. (**A)** Region 1 comprises a (rare) H1 single peak (a) and two L2 peaks (b,c). Region 3 comprises three L2 peaks (d–f); peak d may be seen as a (rare) L1 single peak if the “extended” GC-range of isochores is used (see Supplementary Table S1). (**B)** Regions 4 and 5 comprise the multi-peak H1 and H2 isochores respectively. (**C**) Region 6 comprises H3 peaks, region 2 corresponds to a compositionally even L1 isochore.

The compositional point-by-point profile (split into several regions) of chromosome 21 (see Fig. 1D and its enlarged version, Fig. 2) revealed: (1) several single peaks (a to f) located in regions 1 and 3, as well as a compositionally low-heterogeneity L1 isochore present in region 2 (these features are also seen in Fig. 1A–C); (2) the isochores from H1 (region 4) and H2 (region 5) families that consist of multiple-peaks that emerge from common compositional bases; (3) a series of extremely sharp peaks ranging from 40–45% to 55–60% GC (region 6); these peaks, previously visualized (see Fig. 1B,C) as a series of alternating H2 and H3 isochores (because the compositional approach used involved merging into isochores 100Kb segments comprised within the ranges of Supplementary Table S1) should now be called “H3 peaks” since they cannot be strictly defined as H3 isochores not being “fairly homogeneous” sequences.

It so happens that, in contrast with other chromosomes, chromosome 21 barely comprises homogeneous L2 sequences (shown in Supplementary Fig. S2 and in ref.^16^) that define an L2^−^ sub-family, as distinct from an L2^+^ sub-family formed by the L2 single peaks of regions 1 and 3 (these peaks are more frequent in chromosome 21 than in other chromosomes). Indeed, only one example of an L2^−^ isochore is present in chromosome 21, the Y “valley” isochore (see Figs 1D and 2). Moreover, upon close inspection, the peaks of regions 4 to 6 were seen to correspond to the minute peaks of Fig. 1C. This observation is very relevant in that it allows generalizing the present results to all human chromosomes as investigated by the sliding window approach.

**Figure 3:**
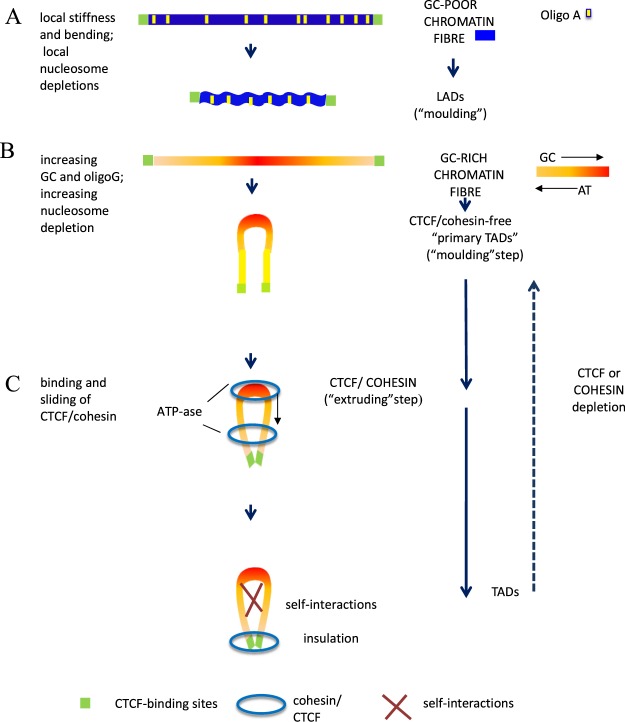
The proposed models for the formation of LADs and TADs. (**A**) A representation of a chromatin fibre corresponding to a GC-poor isochore (a blue bar with yellow oligo-Adenines blocks is delimited by CTCF binding sites, green boxes). Topologically, the structure of the chromatin fibre is modeled by intrinsically stiff and curved oligo-Adenines that cause local bending, twisting and nucleosome depletions; this chromatin structure favors its 3-D physical adaptation to the lamina and may be considered as the result of a “moulding” of LADs by the DNA sequences. (**B**) A compositional representation of a chromatin fibre corresponding to a GC-rich peak; the yellow to red color gradient indicates the corresponding gradients of GC, oligo-Guanines, CpG’s and CpG islands that alternate with the AT gradients of the oligo-Adenines present in the troughs^23^ both gradients being responsible for the decreasing nucleosome density, decreasing supercoiling and increasing accessibility as well as for the looping; this “moulding step” by the DNA sequence is followed by: (**C)** the binding of CTCF and cohesin to the peaks of the loops and by a “sliding/extrusion step” down the loops (helped by the cohesin ATP-ase) which ends when the CTCF/cohesin complex reaches the CTCF binding sites at the base of the loops; this process leads to approaching the two branches of the loop, creating self-interactions and closing and insulating the loop. The broken arrow indicates that the depletion of CTCF or cohesin reverses TADs into “primary TADs”, that still are transcriptionally functional^31–33^.

In conclusion, the point-by-point approach has revealed (1) a low-heterogeneity L1 sequence; (2) several L2^+^ single peaks (in addition to a rare H1 single peak; see legend of Fig. 2); (3) the multi-peak structures of H1 and H2 isochores; and (4) the H3 peaks.

### Oligo-nucleotides and isochores

The compositional and topological properties of isochores will now be considered in more detail. Using previous data^22^, one can assess not only the levels of AAA/TTT, GGG/CCC, but also those of “A/T-only” and “G/C-only” trinucleotides, in isochore families. The results for L1 and H3 isochores, presented in Supplementary Table S2, indicate that specific sets of trinucleotides have widely different distributions in different isochore families. Indeed, in L1 isochores (35.5% GC) the “A/T-only” trinucleotides represent 26% of the sequences, with AAA/TTT corresponding to 9% and consisting of a series of blocks that may extend to tetra- to octa-A’s^23^. In contrast, the L2^+^, H1, H2 and H3 peaks are characterized by gradients of increasing GC and increasing frequencies of oligo-G’s. In the “classical” H3 isochores (55% GC), “G/C-only” tri-nucleotides represent 15.4% of the sequences with GGG/CCC corresponding to 6.2% and extending to tetra- to octa-Gs^23^.

One should now consider that the interspersed oligo-A’s and oligo-G’s are intrinsically stiff for different structural reasons^24^ and that, specifically, oligo-A’s are intrinsically curved^25^. Two different classes of isochores can, therefore, be distinguished from a topological viewpoint: (1) the L1 (and, probably L2^−^) isochores characterized by low compositional heterogeneity that are locally stiff because of the presence of interspersed oligo A’s; and (2) the L2+ and H3 single-peaks and the H1/H2 multi-peaks that are characterized by a high compositional heterogeneity, by increasing densities of interspersed oligo-G’s (as well as of CpG’s and CpG islands^26^), and by increasing densities of oligo-A’s in the troughs of H3 peaks. In conclusion, the A- and G-oligonucleotides are responsible for specific topological differences in GC-poor and GC-rich isochores. Expectedly, “A/T-only” and “G/C-only” sequences also contribute to such topological differences^23^.

### Isochores and nucleosomes

A crucial point now is to consider that the features just described that are responsible for the topology of GC-poor and GC-rich isochores, namely the presence of interspersed oligo-As and oligo-Gs (as well as of CpGs and CpG islands) are strongly inhibitory to nucleosome formation^24–26^. It should also be noted that the 3-D structures of GC-poor and GC-rich isochores appear after nucleosomes dissociate from the parental DNA strands and before they re-associate (along with neo-synthesized nucleosomes) on the daughter strands (see Supplementary Fig. S3A and refs^27,28^).

### Isochores and chromatin domains

Yet another, critical, way to classify isochores can be based on their correspondence with chromatin domains. This also leads to visualize the same two groups of isochores. Indeed, the L1 and L2^−^ isochores (that include some “valley” isochores such as X and Y of Figs 1D and 2) correspond to LADs, while the single-peak and the multi-peak isochores of the other families correspond to interLADs (single-loop and multi-loop TADs, respectively; see Supplementary Fig. S2). In the case of L2 isochores, the existence of two sub-families L2^−^ and L2^+^ is confirmed by their correspondence with LADs and interLADs (*i*.*e*., TADs), respectively (see Supplementary Fig. S2).

### A model for the formation of chromatin domains

The results presented so far allow building models for the formation of LADs and TADs. In the case of LADs (Fig. 3A), the presence of interspersed oligo-A’s that causes local DNA stiffness and bending also causes local nucleosome depletions that induce LADs to assume a structure which is well suited to attach to (and even embed in) the lamina^29^. This structure can also be visualized as the result of a “moulding”of LADs by the short-sequence designs of L1 and L2^−^ isochores.

In the case of TADs, their formation appears to take place through a two-step mechanism. In a critical first step, the increasing oligo-Gs, CpGs and CpG islands of the peaks are responsible for a decreasing nucleosome density^25,26^, a decreasing supercoiling^30^ (and an increasing nuclease accessibility), that constrain chromatin to fold into loops, the tips of the loops corresponding to the highest GC levels and to the lowest nucleosome density. Incidentally, the nucleosome-depleted troughs between the peaks due to the increasing presence of oligo-Adenines help the folding of the chromatin fibre in the valleys between the H3 peaks. This initial “moulding step”, which may involve essentially equal-size chromatin branches (as suggested by the symmetry of GC peaks; see Figs 1D and 2), leads to CTCF/cohesin-free “primary TADs” (Fig. 3B), that probably are already functional since cohesin and CTCF depletions are not followed by any remarkable alteration of transcription as recently shown^31–33^. Interestingly, a wider nucleosome spacing and higher flexibility of the loops compared to boundary regions was proposed by the “insulation/attraction model”^17^.

Two points should now be raised about the “moulding step”. The first one concerns which one of the two factors, the 3-D structure of isochores or the nucleosome density, is primarily responsible for moulding. The demonstration (see Supplementary Fig. S3B) that TADs are not very different in fibroblasts and in spermatozoa^34^ (in which case nucleosomes are replaced by protamines) provides an answer in favor of the first possibility. The second point is that the existence of “primary TADs” helps to solve the notorious open problem of the energy source required by the extrusion models^35,36^, that involve pulling megabase-size chromatin fibres through the CTCF/cohesin ring. Indeed, the “marginal” energy burden proposed in a very recent article^37^ essentially rests on indirect evidence, namely on the fact that an ATP hydrolysis dependent molecular motor was recently identified in condensin and shown to use 2ATP per sec per condensin^38^.

In a second step (Fig. 3C), one may consider that the CTCF/cohesin ring complex binds to the peaks of the loops and starts an “extrusion step” down the “primary TADs”. In this connection, it is of great interest to consider the recent proposal^39^ that Nipbl, an essential factor for loading cohesin on the loops, may also stimulate cohesin translocation down the loops. The ATPase activity associated with cohesin sub-units Smc1 and Smc3, also stimulated by Nipbl^40^, would provide the energy necessary for sliding, an energy obviously far below the level required by “active extrusion”. This sliding step leads to closer contacts between the two branches of the loops and to self-interactions and stops at the CTCF binding sites, that are located at the base of the loops, thus closing and insulating them. Incidentally, the possibility that extrusion could also be mediated by a single cohesin ring sliding over the top of a preformed chromatin loop was taken into consideration previously^41^.

## Conclusions

The present investigations led to (1) the discovery of the 3D topology of isochores; (2) the discovery that this topology is due to short sequences, such as oligo-Adenines and oligo-Guanines, as well as to a number of other short sequences^23^; and (3) the discovery that the 3D topology of isochores is responsible for the “moulding step” of chromatin domains. Incidentally, the roots of the first two conclusions go very back in time^2,3^.

These conclusions represent a paradigm shift compared to current models that only rest on architectural proteins, such as CTCF and cohesin, neglecting, as also remarked by other authors^42^, the role played by DNA. Indeed, they introduce a primary “moulding step” based on isochore topology which precedes the “extrusion step” and makes it easier in terms of the energy required.

These investigations, that only dealt with the evolutionary conserved features of chromatin architecture, also led to a new vision of the “genomic code”, a definition which was originally coined^43,44^ (see also ref.^11^ for a review) for the compositional correlations that hold between coding and contiguous non-coding sequences and among the three codon positions. In fact, the “genomic code” should now be visualized (G. Bernardi, paper in preparation) as (1) a short-sequence code for the 3D topology of isochores and the moulding of chromatin domains; (2) a pervasive code since it not only concerns the ~98% of non-coding sequences, leaving little or no room for “junk DNA” (see ref.^45^ for a review), but also constrains the genetic code in terms of composition of coding sequences, codon usage and aminoacid choice^11^.

## Materials and Methods

The compositional profile displayed in Fig. 1A (from ref.^46^) concerns DNA sequences derived from release hg 38. Non-overlapping 100 Kb windows were used because 100Kb is a plateau value under which the composition of DNA segments shows an increasing variance with decreasing size, due to the contribution of different specific sequences, such as interspersed repeated sequences^10^. Figure 1B shows an isochore profile obtained by using a sliding window approach^16^. Figure 1B,C are from the matched b37 assembly of ref.^47^; these profiles are slightly more extended on the centromeric (left) side than that of Fig. 1A.

### Nomenclature

Although TADs comprise, by definition, all the topologically associating domains, in the present context TADs indicate the chromatin domains other than LADs. The main reason for this choice is that the mechanisms of formation of the two sets of domains are different, even if based on the same DNA properties, 3-D structure of isochores and nucleosome binding.

## Electronic supplementary material

Supplementary Materials

## Data Availability

All data generated or analyzed during this study are included in this published article (and its Supplementary Information files).
