# Supplementary material for: The formation of chromatin domains involves a primary step based on the 3-D structure of DNA
Source: Sci Rep. 2018 Dec 13;8:17821. doi: 10.1038/s41598-018-35851-0 (PMC6292937; doi:10.1038/s41598-018-35851-0)
Supplement: Supplementary file 1 — Supplementary Materials [file 41598_2018_35851_MOESM1_ESM.docx]

**The formation of chromatin domains**

**involves a primary step based on the 3-D structure of DNA**

Giorgio Bernardi

**Supplementary Materials**

- Supplementary Tables S1 and S2
- Supplementary Figures S1 to S3
- Legends of Supplementary Figures
- References

**Supplementary Table S1**

| **ISOCHORE FAMILIES AND CHROMATIN DOMAINS IN THE HUMAN GENOME** | | | | | | | | | | |
| --- | --- | --- | --- | --- | --- | --- | --- | --- | --- | --- |
|  | % | GC  max | GC  min  37  41  46  53 | “Fixed”  GC ranges(a) | “Extended”  GC ranges(a) | Isochore  peaks(b) | Chromatin domain | Chromatin  loops (b) | Genome  % | |
| L1 | 19 | 35.5 |  | 33-37 | 🡪37.6 | n | LADs | n | 19 | 35 |
|  |  |  |  |  |  |  |  |  |  |  |
| L2 | 36 | 38.7 |  | 37-41 | 36.4-42 | n  s | LADs L2^-^(c)  TADs L2^+^(c) | n  s | 16 |  |
|  |  |  |  |  |  |  |  |  | 20 |  |
| H1 | 31 | 43.0 |  | 41-46 | 39.6-46.9 | m | TADs (d) | m | 31 |  |
|  |  |  |  |  |  |  |  |  | 65 | |
| H2^(e)^ | 11 | 48.5 |  | 46-53 | 45-54 | m | TADs | m | 11 |  |
|  |  |  |  |  |  |  |  |  |  | |
| H3^(e)^ | 3 | 55.0 |  | 53-59 | 52🡪 | s | TADs | s | 3 |  |

(a) “Fixed” ranges of the isochore families are those used so far. “Extended” ranges avoid the assignment of an isochore to a family when slightly trespasses its range borders (see Fig.2 for examples).

(b) n, no peak or no loop; s, single peak or single loop; m, multiple-peaks or multiple-loops. Multi-peak isochores are much more frequent than single-peak isochores as judged from previous results and by the fact that H1+H2 isochores (largely corresponding to multiple peaks) represent ~42% of the genome *vs* ~23% for L2^+^ + H3 isochores (largely corresponding to single peaks).

(c) L2^-^ isochores are even or “valley” isochores; they can be estimated to represent ~16% of the genome, since all LADs represents ~35% and L1 isochores 19%. Likewise, L2^+^ single-peak isochores can be estimated at 20% of the genome by the difference between 65% (all TADs) and 45% (H1, H2 and H3).

(d) The contribution of H1 to LADs is very small.

(e) These data correspond to “classical” H3 isochores as defined by the compositional approach used so far, in which 100Kb sequences are assembled into isochores according to their composition. Using 100Kb sequences in point-by-point plots leads to the definition of “H3 peaks” (see text).

**Supplementary Table S2**

**TRI-NUCLEOTIDE DISTRIBUTION (%)**

**IN ISOCHORE FAMILIES**

|  | **L1^(a)^** | **H3^(b)^** | **L1/H3^(c)^** | **H3/L1^(c)^** |
| --- | --- | --- | --- | --- |
| “A/T-only”  tri-nucleotides ^(d)^ | 26 | 9.0 | 2.9 | 0.35 |
| AAA/TTT^(e)^ | 9 | 4 | 2.25 | 0.44 |
| “G/C-only”  tri-nucleotides ^(d)^ | 4 | 15.4 | 0.26 | 3.9 |
| GGG/CCC^(f)^ | 1.8 | 6.2 | 0.29 | 3.4 |
| AT | 64.5 | 45 | 1.43 | 0.78 |
| GC | 35.5 | 55 | 0.64 | 1.55 |

(a) The situation found in L1 isochores is most likely to be present, even if slightly less

pronounced, in L2^-^ isochores since in the whole L2 family “A/T-only” tri-nucleotides correspond

to 20% of the total, a value not too far from 26% in L1 isochores.

(b) Values concern “classical” H3 isochores (see Supplementary Table S1).

(c) These ratios are shown to stress the large difference with the corresponding GC ratios of

isochore families (bottom lines).

(d) The ratio of “G/C-only” / “A/T-only” tri-nucleotides between H3 and L1 isochores is equal to

11 vs. a GC/AT ratio of only 1.55.

(e) AAA/TTT may extend to tetra octa-A not only in L1/LADs but also in the troughs that

separate the H3 peaks (G.Lamolle, V.Sabbia, H.Musto, G.Bernardi, paper in preparation).

(f) GGG/CCC may extend to tetra octa-G in the L2+, H1, H2, H3 peaks.

L2

Mb


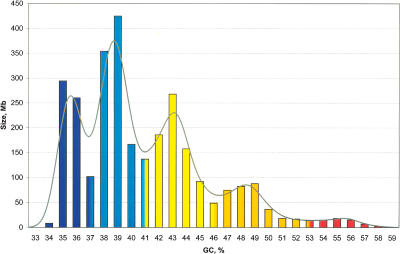


**Genome desert ~35%**

H3

H2

H1

L1

GC

**Genome core** ~65%

GC-rich

Gene-rich

Open chromatin

Early replicating

Fig. S1 (Bernardi)


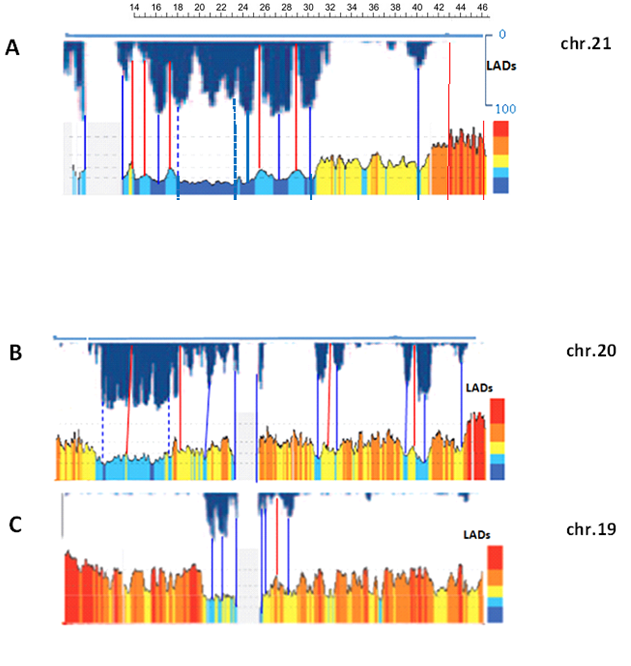


**Mb**

Fig.S2 (Bernardi)

**
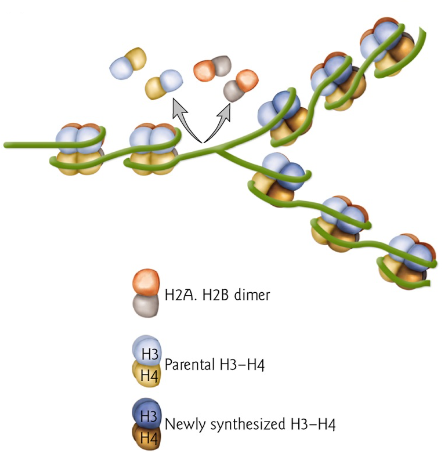
**

A

**
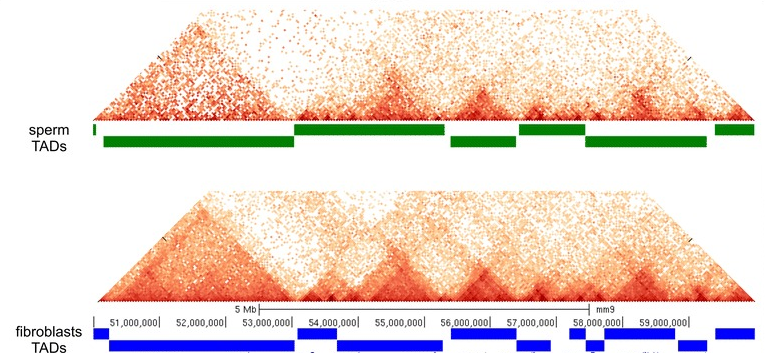
**

B

Fig.S3 (Bernardi)

**Supplementary Fig. S1. Isochore families.** The histogram (modified from ref. 1) displays the isochores from the human genome as pooled in bins of 1% GC. The Gaussian profile shows the distribution of isochore families, that are represented in different colors (color code as in Fig.1 of main text). Gene densities define a “genome desert”, comprising isochore families L1 and L2^-^ , and a “genome core”, comprising isochore families L2^+^ ,H1, H2, H3 (L2^-^ and L2^+^ subfamilies are separated by a vertical broken red line). Permission to publish this figure was obtained from the copyright owner.

Originally, the five isochore families of the human genome were divided on the base of GC levels and gene concentration into GC-poor L (L1+L2), GC-rich H1 and H2 and GC-richest H3 families (the latter being called the “genome core”^2^ ). Subsequently^3^ : 1) the H2 isochores were included in the “genome core”;2) L1 and L2 isochores were called “the empty space” or the “genome desert”; and 3) H1 isochores were assigned to the “genome desert” because of an underestimate of gene density in H1 isochores in the gene set used (9,315 genes from ref.4); this bias was absent in a larger set of 19,702 genes ^5^ and led to the present re-inclusion of H1 isochores in the “genome core” which should now also comprise the L2^+^ subfamily.

**Supplementary Fig. S2. The isochore profiles of human chromosomes** (modified from ref.6) are compared with the inverted LAD profiles^7^ . The multicolored bars on the right are the color codes for isochore families. **A.** The isochore profile of chr. 21 shows the correspondence of LADs 1) with L1 isochores (blue lines; two broken blue lines bracket the largest L1 isochore, and 2) with two “valley” isochore (blue lines, X and Y); two LADs, last on the left, flank the centromere; one H1 and five L2^+^ “peak” isochores (red lines) correspond to interLADs/ TADs. The Mb scale on the top concerns chromosome 21; the same-spacing scale was used for chromosomes 20 and 19. **B.** The isochore profile of chr. 20 shows the correspondence of LADs with 1) a large flat L2^-^ isochore (bracketed by broken blue lines; on the left of the panel) which includes an interLAD (red line); and 2) with several L2^-^ “valley” isochores (blue lines); interLADs correspond to GC-rich isochores (red lines); two faint LADs (blue lines) flank the centromere. **C.** The isochore profile of chr. 19 shows that two LADs (on the right) correspond to H1 isochores (blue lines; a rare case; see Supplementary Table S1) flanking an H2 isochore, an interLAD (red line); two other LADs (on the left) correspond to L2^-^ “valley” isochores (blue lines); two faint LADs flank the centromere. Permission to publish this figure was obtained from the copyright owner.

**Supplementary Fig. S3.** **A.** **Nucleosome assembly at DNA replication.** The dimeric model: parental H3-H4 tetramers are dissociated into dimers and are paired with newly synthesized H3 H4 dimers on each daughter DNA strand. In this model, H3-H4 dimers from parental nucleosomes are segregated evenly onto daughter DNA strands. (From refs.8 and 9). Permission to publish this figure was obtained from the copyright owner. **B.** **TADs as present in sperm cells and fibroblasts** of a region of chromosome 19. The TAD signal shows visible similarity between sperm cells and fibroblasts. (From ref.10). Permission to publish was obtained from the copyright owner.

References:

1. Costantini, M., Clay, O., Auletta, F., Bernardi, G. An isochore map of human chromosomes. *Genome Res* **16**,536-541 (2006).
2. Bernardi, G. Isochores and the evolutionary genomics of vertebrates. *Gene* **241**,3–17 (2000).
3. Bernardi, G. Misunderstandings about isochores. Part 1. *Gene* **276**,3-13 (2001).
4. International Human Genome sequences Consortium. Initial sequences and analysis of the human genome. *Nature* **409**,860-921 (2001).
5. Jabbari, K., Nürnberg, P. A genomic view on epilepsy and autism candidate genes. *Genomics* **108**,31-33 (2015).
6. Jabbari, K., Bernardi, G. An isochore framework underlies chromatin architecture. *Plos One* http://dx.doi.org/10.1371/journal.pone.0168023 (2017).
7. Kind, J. *et al.* Genome-wide Maps of Nuclear Lamina Interactions in Single Human Cells. *Cell* **163**,134–147 (2015).
8. Tagami, H., Ray-Gallet, D., Almouzni, G., Nakatani, Y. Histone H3.1 and H3.3 complexes mediate nucleosome assembly pathways dependent or independent of DNA synthesis. *Cell* **116**, 51-61 (2004).
9. Allison, L. A., *Fundamental Molecular Biology.* Blackwell (2007).
10. Battulin, N. *et al.* Comparison of the three-dimensional organization of sperm and fibroblast genomes using the Hi-C approach. *Genome Biology* **16**, 77-91 (2015).
